# Clinical outcomes of endovascular aortic repair in blunt traumatic aortic injury: A retrospective case series and a CARE-compliant case report

**DOI:** 10.1097/MD.0000000000048100

**Published:** 2026-03-20

**Authors:** Changbao Yan, Jie Zhang, Dafang Liu, Yanyang Wang, Liang Zhao

**Affiliations:** a Department of Vascular Surgery, Beijing Luhe Hospital, Capital Medical University, Beijing, China.

**Keywords:** aortic dissection, aortic endovascular exclusion, stent implantation, surgery, trauma

## Abstract

**Rationale:**

Blunt traumatic aortic injury (BTAI) is a high-mortality condition often managed by endovascular aortic repair (EVAR) due to its minimally invasive nature and favorable outcomes.

**Patient concerns:**

Six patients with BTAI were admitted to our institution from 2014 to 2023. They presented with symptoms such as chest pain or were diagnosed with aortic injury on chest computerized tomography after motor vehicle collisions.

**Diagnosis:**

All patients underwent computed tomography arteriography to confirm the diagnosis and assess the aortic injury.

**Interventions:**

All patients received EVAR. Among these cases, 5 were classified as grade II and 1 was classified as grade IV. The patient with grade IV injury and intraoperative shock died of respiratory failure postoperatively. The remaining 5 patients had favorable outcomes.

**Outcomes:**

The mean follow-up was 3 years. No recurrent chest pain, spinal cord ischemia, or other aorta-related complications were observed. One patient experienced a pulmonary embolism and another had a cerebral infarction, both managed conservatively.

**Lessons:**

EVAR is effective for BTAI, especially in reducing early mortality and complications. Careful evaluation of comorbidities and associated injuries is crucial, particularly in older patients.

## 1. Introduction

Traumatic aortic injury (TAI) has been documented and managed for centuries, with the earliest documented case reported by Vesalius in 1557, describing an aortic injury resulting from a fall from horseback.^[1]^ Autopsy studies of individuals who experienced traumatic injury have indicated that aortic rupture occurs in 12% to 23% of cases, ranking second only to blunt injury as a cause of mortality in 1% of fatal brain injuries.^[1]^ The prehospital mortality rate is reported to be 85%, with most individuals succumbing to their injuries without receiving effective treatment following hospital admission.^[2,3]^ Blunt traumatic aortic injury (BTAI) represents a subset of TAI and is classified into grades I, II, III, and IV (grade I is intimal tear; grade II is intramural hematoma; grade III is pseudoaneurysm; and grade IV is rupture) based on aortic injury grading criteria.^[4]^ Surgical intervention remains the primary therapeutic approach for effective management for grades II to IV, while grade I (intimal tear) injuries are typically managed conservatively.^[5,6]^ Currently, endovascular aortic repair (EVAR) is recommended as the preferred treatment modality for BTAI due to its associated benefits in survival rates and reduced incidence of complications.^[7]^

BTAI refers to structural damage to the aorta resulting from high-energy external forces, such as those sustained in motor vehicle collisions or falls. This condition most commonly affects the aortic isthmus. Although its incidence is relatively low, BTAI is associated with a high fatality rate, with prehospital mortality reported in 75% to 90% of cases. Traditionally, management has involved thoracotomy repair combined with artificial vascular grafting; however, this approach is associated with significant surgical trauma and a high risk of complications, including paralysis secondary to spinal cord ischemia. In recent years, EVAR has increasingly been utilized as a primary treatment modality due to its minimally invasive nature and rapid recovery. However, further investigation is required to assess its long-term efficacy, broaden its indications, and optimize management strategies for individuals with complex aortic anatomies.

Previous research has demonstrated that EVAR is associated with favorable clinical outcomes and a favorable safety profile in the management of acute aortic dissection (AD). However, limited clinical experience exists regarding its application BTAI. Individuals with BTAI frequently present with multiple concomitant injuries and severe physiological compromise, making them less tolerant of open surgical repair due to its invasive nature and associated surgical trauma. This study focuses on evaluating EVAR as a treatment modality for BTAI, with the objective of reducing early mortality and complication rates.

Since the establishment of the Traffic Accident Treatment Center at this hospital, >4000 trauma cases have been treated annually. Since 2014, 6 individuals diagnosed with BTAI have received treatment, with an incidence rate of 2.3 per 100,000. Among these cases, 5 were classified as grade II and 1 was classified as grade IV according to the aortic injury grading system. All aortic injuries were located distal to the left subclavian artery (LSA). Currently, no standardized classification system exists for the anatomical location of BTAI. To facilitate the development of surgical strategies, classification criteria for AD were referenced, and these injuries were categorized as Stanford type B.^[8]^ All individuals in this study underwent EVAR.

The aim of this study was to analyze the management and outcomes of individuals diagnosed with BAI at this center, including admission, treatment, and follow-up. By summarizing both successful outcomes and challenges encountered, the aim of this study was to provide a reference for clinical decision-making and treatment strategies.

The pathological mechanisms underlying BTAI involves the “water hammer effect” and the sterno-spinal compression theory, both of which contribute to intimal tearing at the aortic isthmus or the formation of a pseudoaneurysm. These pathological changes share similarities with the etiology and progression of acute AD. The theoretical advantage of EVAR lies in its use of a stent graft to exclude the site of rupture, restore blood flow within the true lumen, and minimize surgical trauma. Consequently, the application of EVAR for the management of BTAI aligns with established clinical diagnostic and treatment modality principles, both in theory and in practice.

## 2. Case data

Between January 2014 and December 2023, 6 individuals diagnosed with BTAI were admitted to this center. All cases were classified as aortic injury grade II or IV and corresponded to Stanford type B. Following evaluation, all individuals underwent EVAR. Inclusion criteria consisted of: diagnosis of BTAI classified as aortic injury grades II, III, or IV and Stanford type B, stable vital signs, deemed suitable for surgical intervention, and absence of absolute contraindications.^[9]^ Exclusion criteria included: lesions located in the ascending aorta or aortic arch, unsuitability for endovascular therapy, unstable vital signs, inability to tolerate surgery, and presence of severe injuries or other comorbid conditions. This study was conducted in accordance with the declaration of Helsinki. This study was conducted with approval from the Ethics Committee of Capital Medical University Affiliated BeiJing Luhe Hospital (KJ2021CX009-26). A written informed consent was obtained from all participants.

The baseline characteristics of the 6 individuals included in the study are summarized in Table 1. The cohort consisted of 3 male and 3 females, with ages ranging from 23 to 68 years and a mean age of 47.5 years. All injuries resulted from motor vehicle collisions. Three individuals presented with persistent chest pain, while the remaining 3 had findings indicative of aortic injury based on chest computed tomography (CT). No complications were observed in individuals younger than 35 years. In contrast, all individuals older than 60 years developed complications, including perioperative respiratory failure requiring endotracheal intubation and ventilator-assisted breathing. All 3 individuals in this age group had a history of hypertension. Additionally, one individual had renal insufficiency and a history of aortic valve replacement. Another individual, classified as Aortic Injury Grading Class IV, had interstitial lung changes and pulmonary bullae and had previously undergone left lung cancer resection. One individual developed cerebral infarction postoperatively. The Injury Severity Score (ISS) among the 6 individuals ranged from 25 to 41, with a mean score of 32.2. All individuals were admitted to the intensive care unit (ICU), and 5 presented in a state of shock upon admission. Three individuals required additional surgical interventions to address associated injuries.

**Table 1:** Patient characteristics.

Patient Number	1	2	3	4	5	6
Gender	Male	Male	Female	Female	Female	Male
Age (yr)	23	33	65	35	61	68
Mechanism of injury	Traffic accident	Traffic accident	Traffic accident	Traffic accident	Traffic accident	Traffic accident
Caution sign	Chest pain	Chest pain	Chest CT	Chest CT	Chest pain	Chest CT
Complicating disease	None	None	Hypertension/aortic valve surgery/renal insufficiency	None	Hypertension/cerebral infarction	Hypertension/left lung cancer surgery/interstitial change/bullae
Complicated injury	Right hemothorax	Multiple rib fractures 1 to 8 on left side*	Multiple rib fractures from 3 to 8 on right side	Pelvic fracture*	Cerebral hemorrhage	Rib fracture (5–8 on left/4–7 on right/left hemothorax)
	Right renal contusion	Left humeral shaft fracture*	Hemopneumothorax/pulmonary contusion	Hemothorax	Left supracondylar fracture of femur*	Subarachnoid hemorrhage
	Surgical neck fracture of right humerus	Pelvic fracture	Pelvic fracture	Contusion/skull base fracture	Rib fracture (6–8 on left/2–7 on right)/pulmonary contusion	Thoracic 12 vertebral fracture
ISS score	29	32	32	41	34	25
Shock	None	Yes	Yes	Yes	Yes	Yes
Respiratory failure/intubation	None	Yes	Yes	Yes	Yes	Yes
Aortic injury grading	II	II	II	II	II	IV

Three patients were treated with surgery to deal with complicated injuries, represented by “*”.

CT = computerized tomography, ISS = Injury Severity Score.

## 3. Treatment

### 3.1. Initial treatment modality

Individuals with an ISS exceeding 16 were admitted to the ICU for close monitoring and management. Those with lower ISS scores were either admitted to a general ward, remained in the emergency department, or were discharged following appropriate treatment. Ongoing observation and reassessment were conducted to inform subsequent clinical decisions. Computed tomography arteriography (CTA) was performed in individuals presenting with persistent chest pain or those with suspected aortic injury on initial CT. CTA was utilized to evaluate the precise location of the aortic tear, its distance from the LSA, the vessel diameter in the anchoring region, lesion length and extent, involvement of splanchnic arteries, and the diameter of access vessels. These measurements were essential for determining the appropriate surgical strategy and intervention approach.

### 3.2. Surgical treatment strategy

All surgical procedures were performed in a catheterization laboratory. General anesthesia was administered to one individual with respiratory failure, while the remaining 5 individuals underwent the procedure under local anesthesia. Intraoperative anticoagulation with heparin was not utilized in any case. Vascular access was obtained via the femoral artery (TERUMO RS*A60K10SQ 6F*) and radial artery (TERUMO RSA50G16SQZ 5F). Initially, a 0.035-inch soft guidewire (TERUMO RFPA35183M/RFGA35263M) was introduced through the brachial artery, followed by an aortogram using a pigtail catheter (Cordis PIG 5F451-503H5) to reconfirm the diagnosis and obtain precise anatomical measurements. This access was subsequently used for placement of the chimney stent. The proximal anchoring site of the stent graft was identified. Next, 2 percutaneous suture devices (Abbott Perclose ProGlide) were prepositioned via the femoral artery. The delivery sheath [GORE DSF(20-24)33] was then exchanged, and a catheter was advanced into the ascending aorta. A stiff guidewire (COOK TSMG-35-260-LES) was introduced, followed by deployment of the stent graft (Table 2) at the target location. Depending on lesion length, 1 or 2 stents were placed. In cases where the distance from the LSA to the lesion was <15 mm, a chimney stent was placed to preserve LSA perfusion.^[10,11]^ In cases of internal hemorrhage, LSA embolization with a coil was performed. Upon completion of the procedure, hemostasis was achieved at the femoral artery puncture site by tightening the suture device, while the brachial artery puncture site was manually compressed for 20 minutes to ensure hemostasis. Once arterial access was confirmed to be free of bleeding, a compression bandage was applied. Postoperatively, all individuals were transferred to the ICU for continuous monitoring and subsequently moved to a general ward upon clinical stabilization.

**Table 2:** Patient treatment and prognosis.

Patient Number	1	2	3	4	5	6
Time from injury to operation (h)	10	42	104	14	30	10
Duration of operation (min)	90	115	80	38	40	65
Tear site	Lesser curvature	Greater curvature	Lesser curvature	Lesser curvature	Lesser curvature	Greater curvature
Distance from LSA to tear (mm)	23	70	20	10	10	0
Range of lesion (mm)	20	0	240	40	25	60
Diameter of anchoring area (mm)	22	21	30	28	25	32
Visceral arteries are involved	No	No	Trunk celiac artery	No	No	No
Stent	Ankura®24 × 80	Gore C-TAG26 × 100	Hercules Low Profile 3228160 + 3026160	Hercules Low Profile 3228160	Hercules Low Profile 3026160 + Boston Scientific Express 8 × 37 (Chimney stent of LSA)	Hercules Low Profile 3632160 + COOK MWCE-35-8-10 (Embolotherapy LSA)
Oversize	9%	24%	7%	14%	20%	13%
LSA block	No	No	Portion	No	No	Yes
Prognosis	Well	Well	Left vocal cord paralysis/pulmonary embolism after 2 months later	Well	Cerebral infarction	Death

LSA = left subclavian artery.

### 3.3. Therapeutic effect

All 6 individuals included in this study underwent EVAR, achieving a technical success rate of 100%. Our cardiovascular surgery department mainly performs EVAR surgery and does not perform open surgery. Moreover, the injury to the ascending aorta and arch (Stanford A) was treated by cardiac surgery at our center, while the injury to the descending aorta (Stanford B) was treated by vascular surgery. Detailed procedural data are presented in Table 2. The interval between injury and surgical intervention ranged from 10 to 104 hours, with a mean duration of 35 hours. Surgical duration varied between 38 and 115 minutes, with an average of 71.3 minutes. The rupture site was located on the lesser curvature of the aorta in 4 individuals and on the greater curvature in 2 individuals. The distance from the rupture site to the LSA ranged from 0 to 79 mm, with a mean of 22 mm. Lesion involvement extended between 0 and 240 mm, with an average length of 64 mm. The diameter of the anchoring area ranged from 21 to 32 mm, with a mean of 26 mm. Stent graft oversizing ranged from 7% to 24%, with an average of 14.5%.

One individual demonstrated celiac trunk artery involvement but did not develop splanchnic ischemia. Stent graft selection varied among the cases: 1 individual received an Ankura stent, 1 received a Gore stent, and 4 received Hercules stents. In 1 case, due to the proximity of the rupture site to the LSA, the artery was covered by the aortic stent, necessitating reconstruction using a bare stent. Another individual underwent coil embolization of the LSA due to type Ia endoleak and a rupture in the aortic arch bulge.

No postoperative complications were observed in individuals younger than 35 years. However, among individuals older than 60 years, 1 female developed left vocal cord paralysis and pulmonary embolism, both of which were managed conservatively with favorable recovery. Another female experienced a cerebral infarction, which also resolved with conservative treatment modality. One male individual with a grade IV injury developed persistent intraoperative shock and died of respiratory failure 7 days postoperatively. These outcomes are presented in Table 2.

Following stabilization, individuals were transferred from the ICU to a general ward for postoperative management. Discharge occurred approximately 1 week after achieving clinical stability. Follow-up assessments were conducted at 3 months, as well as at 1, 3, and 5 years postoperatively, including aortic CTA to monitor vascular integrity and treatment durability.

### 3.4. Follow-up

**Figure 1.**
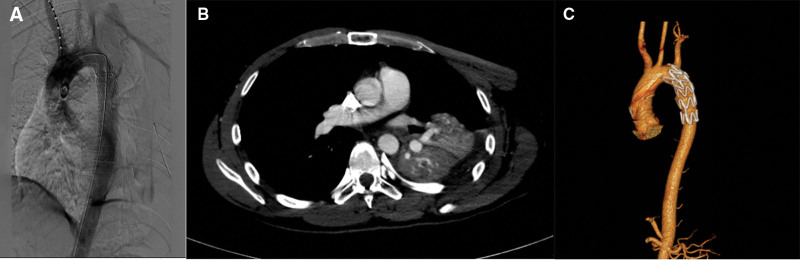
A 35-year-old male with BTAI caused by a car accident. (A) The tear was located at the lesser curvature of descending aorta seen by preoperative CTA. (B) The postoperative reexamination showed that the lesion was well sealed without endoleak, and the LSA had no obstruction. (C) The stent was in good shape and position with no endoleak and no obstruction of the LSA during the follow-up 5 years after the operation. BTAI = blunt traumatic aortic injury, CTA = computed tomography arteriography, LSA = left subclavian artery.

The duration of follow-up ranged from 6 months to 5 years (Fig. 1). Among the 5 surviving individuals, no cases of recurrent chest pain, spinal cord ischemia, left upper extremity ischemia, internal hemorrhage, or other aorta-related complications were reported. One individual presented to the emergency department with acute dyspnea 2 months postoperatively and was diagnosed with a pulmonary embolism. However, vital signs remained stable, and the individual recovered well following anticoagulation therapy. Another individual experienced impaired right limb mobility due to a postoperative cerebral infarction. After 2 months of conservative treatment, limb function improved, though some residual impairment persisted compared to the contralateral limb.

## 4. Discussion

Previous studies have demonstrated that aortic rupture occurs in approximately 12% to 23% of individuals sustaining trauma.^[1]^ TAI is recognized as the second leading cause of trauma-related mortality, with blunt trauma accounting for approximately 10% of all aortic injuries.^[1]^ BTAI is primarily a deceleration injury, commonly resulting from motor vehicle collisions or falls.^[12]^ The injury predominantly occurs at the relatively fixed aortic isthmus. Pathophysiologically, BTAI is shares similarities with nontraumatic AD.^[13]^ Consequently, EVAR has been increasingly adopted as a treatment strategy for BTAI.

Between January 2014 and December 2023, 6 patients with BTAI were admitted and included in this study, resulting in an incidence rate of 2.3 per 100,000 trauma cases. This data reflects the local prevalence of BTAI in our institution, which is lower than that reported in the literature.^[14,15]^ Among the 6 included patients, 1 patient with grade IV injury died of respiratory failure 7 days postoperatively, resulting in an in-hospitalmortality rate of 16.67%. This relatively high rate may be attributed to the small sample size and the inclusion of a grade IV rupture case with severe comorbidities (e.g., interstitial lung disease, prior lung cancer resection) and intraoperative shock, which significantly increased the risk of adverse outcomes. Additionally, as noted in the manuscript, BTAI is associated with a high prehospital mortality rate,^[2,3]^ but our data focuses on in-hospital mortality among patients who survived to receive treatment, highlighting the critical role of timely intervention such as EVAR in improving survival for those who reach the hospital. Only Stanford type B BTAI was identified in this study cohort, which may be attributed to the inclusion criteria. Individuals who succumbed at the scene of injury or those who died shortly after admission to the emergency department without a confirmed diagnosis were excluded from the analysis.

Several mechanisms have been proposed to explain the pathophysiology of BTAI: excessive traction on the aortic ligament due to the relative fixation of the aortic isthmus; a sudden and severe increase in blood pressure; aortic rupture secondary to thoracic spine compression; and the “water hammer effect,” which results from the abrupt cessation of blood flow.^[16]^ These mechanisms collectively account for the majority of aortic injuries encountered in clinical settings.

**Figure 2.**
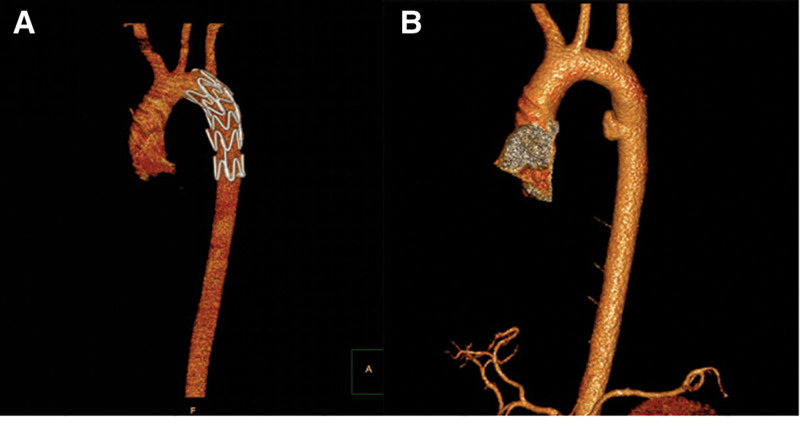
Multiple rib fractures in a 32-year-old male from a car accident injury. (A) Fracture of the end of the 4th posterior rib lead to the descending aorta injury. (B) EVAR was performed, followed by rib reduction and internal fixation. EVAR = endovascular aortic repair surgery.

A unique case was identified in this study in which aortic injury was attributed to the displacement of a fractured segment of the left 4th posterior rib, which punctured the aorta. This mechanism, although uncommon, may represent a novel etiology of aortic injury. Despite the absence of significant contrast extravasation on CTA of the aorta, the diagnosis was supported by evidence of aortic compression and the sharp configuration of the rib fracture. The management strategy involved an initial EVAR procedure to ensure vascular stabilization, followed by rib reduction and internal fixation to prevent further injury (Fig. 2). This case underscores the necessity of considering posterior rib fractures as a potential cause of aortic injury. Given the anatomical proximity of the descending aorta on the left side of the thoracic vertebrae, and the tendency for anterior displacement of the 9th rib in relation to the thoracic spine, individuals with fractures of the left 1st through 8th ribs warrant vigilant monitoring. Prompt diagnosis with aortic CTA is crucial to facilitate timely intervention and prevent catastrophic complications.

All individuals in this study underwent intervention in accordance with the principles outlined in the Advanced Trauma Life Support.^[9]^ The ISS among individuals with BTAI ranged from 25 to 41, with a mean of 32.2, which closely aligns with the 32.5 reported in previous literature.^[17]^ Given the high incidence of polytrauma in these cases, symptoms associated with BTAI are often overlooked. In this study, 3 individuals reported persistent chest pain, whereas the remaining 3 were diagnosed with aortic abnormalities based on findings from an initial chest CT scan. In individuals presenting with severe trauma, particular attention should be given to the presence of chest pain and chest CT findings indicative of peri-aortic hematoma or effusion. Additional diagnostic indicators include mediastinal widening observed on chest radiography; however, this finding has limited sensitivity and specificity, reducing its utility in clinical practice.

CTA remains the primary imaging modality recommended by BTAI guidelines, as it offers sensitivity and specificity between 98% and 100% and a negative predictive value approaching 100%.^[18–20]^ Transesophageal echocardiography demonstrate sensitivity and specificity ranging from 97% to 100% and 98% to 100%, respectively; however, it is not widely performed due to technical limitations in many clinical settings.^[21]^ Magnetic resonance angiography provides diagnostic accuracy comparable to that of CTA but requires an extended examination time, making it less suitable for acute trauma evaluation.^[22]^ Nonetheless, magnetic resonance angiography may serve as a viable alternative for follow-up imaging, particularly in individuals with renal impairment or contraindications to iodinated contrast agents.^[1]^

BTAI is associated with a high fatality rate, with 32% of individuals succumbing within the 1st day of hospitalization, 61% within the 1st week, and 74% within the 2nd week.^[23]^ Additionally, 30% of individuals who survive the acute phase without surgical intervention are at risk of developing advanced traumatic thoracic aortic aneurysm, which progress to rupture.^[24]^ Given this high risk, early surgical intervention is strongly recommended.^[7,9]^

Historically, open surgical repair has been the primary treatment modality for traumatic aortic injuries, since Pasaro first performed a successful open surgery for traumatic AD in 1959.^[25]^ However, the extended procedural duration, significant surgical trauma, and high incidence of perioperative complications associated with open surgery present considerable challenges.^[5,26]^

Since the introduction of artificial stent grafts for BTAI by Santanello in 2002, EVAR has gained widespread acceptance due to its minimally invasive nature, reduced complication rates, and improved survival outcomes.^[6,27,28]^ Given the recent advancements and positive clinical outcomes, EVAR has emerged as a safer and more effective treatment modality and endorsed in vascular surgery guidelines since 2011.^[7]^ In the present study, all 6 individuals with BTAI underwent EVAR, achieving a 100% technical success rate. Although one individual with grade IV BTAI succumbed to respiratory failure postoperatively, the remaining individuals exhibited favorable recovery outcomes. The longest follow-up period extended to 5 years, during which no aorta-related complications (such as internal hemorrhage or stent displacement) were observed. These findings further support the safety and efficacy of EVAR in the management of BTAI.

The mean time from injury to surgical intervention in the present study was 35 hours, which is shorter than the reported average of 39.2 hours in similar Asian populations.^[17]^ Additionally, the average surgical duration was 71.3 minutes, with the shortest procedure completed in 38 minutes (significantly lower than the regional average of 100 minutes).^[17]^ These findings suggest a more rapid institutional response and reduced procedural time, both of which are critical in individuals with severe trauma, as early intervention and shortened operative duration are key prognostic factors. EVAR generally requires a proximal anchor zone of at least 15 mm to ensure optimal stent fixation.^[7]^ In the current cohort, the mean anchor zone length was 22 mm. However, 2 individuals presented with anchor lengths of only 10 mm. One individual underwent LSA chimney stent placement, with the anterior edge of the aortic stent released near the posterior edge of the LSA. In another individual, partial coverage of the LSA was performed without subsequent evidence of internal hemorrhage or organ ischemia. One individual had an anchor area length of 0 mm, necessitating LSA embolization to expedite the procedure due to the severity of the condition. However, this individual ultimately succumbed to postoperative respiratory failure. Given that individuals with BTAI often sustain severe injuries, prolonged and complex surgical procedures may not be feasible. Furthermore, most rupture sites are located along the lesser curvature of the aortic arch. Studies have demonstrated that LSA coverage is generally well tolerated and rarely necessitates secondary reconstruction.^[29,30]^ Consequently, a shorter anchor area requirement, strategic LSA closure when necessary, and rapid rupture control may contribute to improved prognoses. When ischemic complications do occur, delayed LSA revascularization can be performed once the individual is clinically stabilized. Notably, no complications related to LSA coverage were observed in the present study.

In the present study, lesion lengths associated with BTAI ranged from 0 to 240 mm, with an average length of 64 mm. Notably, no cases of organ ischemia were observed following surgical intervention. Most lesions were relatively short and did not involve internal supplying arteries, allowing for effective stent placement without compromising blood flow. Given that successful EVAR depends on adequate tear coverage, proper stent selection is crucial to preventing complications such as internal hemorrhage. Previous studies have identified internal hemorrhage as the most prevalent complication following EVAR for BTAI, with an estimated incidence of approximately 14.4%.^[31]^ To mitigate such risks, selecting an appropriately sized aortic stent is essential, as improper sizing may lead to complications such as hemorrhage, stent collapse, or stent displacement. Current practice guidelines recommend an oversizing range of 10% to 20% relative to the aortic stent diameter.^[31,32]^ In this study, the aortic anchor zone diameter ranged from 21 to 32 mm, with a mean diameter of 26 mm. The degree of stent oversizing varied from 7% to 24%, with a mean oversizing of 14.5%. Importantly, no stent-related complications, including internal hemorrhage, were observed. These findings underscore the importance of precise stent selection and adherence to recommended oversizing parameters in optimizing clinical outcomes for individuals undergoing EVAR for BTAI.

Previous studies have reported a combined morbidity rate of 2.2%, with all-cause and aorta-related mortality rates of 2.1%.^[17]^ However, the present study observed a perioperative complication rate of 50%, with all-cause and aorta-related mortality rates at 16.67%. These differences may be attributable to the small sample size in this study.

A notable finding was the absence of complications or mortality among patients younger than 35 years. Conversely, all individuals aged over 60 years developed postoperative respiratory failure. Among these, one individual experienced vocal cord paralysis, another developed cerebral infarction, and one patient died. No cases of internal hemorrhage or renal function deterioration were observed. These findings indicate that advanced age may be a significant factor contributing to poorer prognoses in individuals with BTAI. Additionally, one patient with a history of lung cancer surgery, concomitant lung disease, multiple rib fractures, subarachnoid hemorrhage, and thoracic vertebrae fractures ultimately succumbed to respiratory failure. This underscores the importance of assessing comorbid injuries and cardiopulmonary function in patients with multiple traumas. Another patient developed a pulmonary embolism 2 months postoperatively. Although successfully treated, this event highlights the need for improved long-term follow-up protocols in patients with BTAI and multiple injuries. Furthermore, these findings underscore the importance implementing enhanced preventive strategies for venous thromboembolism in this patient population.

## 5. Conclusion

BTAI is associated with a high mortality rate and requires timely and effective intervention. Although the sample size in the present study was limited, the findings indicate that EVAR experienced good outcomes due to its minimally invasive nature, reduced operative duration, and lower incidence of complications. Effective management of BTAI extends beyond addressing the primary vascular injury and should include thorough evaluation of comorbid conditions and associated injuries. Advanced age appears to be a significant prognostic factor for adverse outcomes. Furthermore, individuals presenting with posterior rib fractures and asymptomatic individuals may harbor clinically significant aortic injuries. Such cases should therefore be carefully evaluated to ensure timely diagnosis and improve prognostic outcomes.

## Author contributions

**Conceptualization:** Changbao Yan, Liang Zhao.

**Data curation:** Changbao Yan, Jie Zhang, Dafang Liu, Yanyang Wang.

**Formal analysis:** Changbao Yan, Jie Zhang, Dafang Liu, Yanyang Wang, Liang Zhao.

**Writing – original draft:** Changbao Yan, Dafang Liu.

**Writing – review & editing:** Changbao Yan, Jie Zhang, Liang Zhao.

## References

[1] Vesalius ABT. Sepulchretum sive anatomia practica ex cadaveribus morbo denatis. Sect 2. Leonard Chouet; 1679:290.

[2] Miller LE. Potential long-term complications of endovascular stent grafting for blunt thoracic aortic injury. Sci World J. 2012;2012:897489. 10.1100/2012/897489 PMC3322436 22547999

[3] Wang Y Li T Liu J. Endovascular repair of traumatic aortic dissection: a single-center experience. Rev Cardiovasc Med. 2021;22:1029–35. 34565104 10.31083/j.rcm2203112

[4] Fortuna GJ Perlick A Dubose JJ. Injury grade is a predictor of aortic-related death among patients with blunt thoracic aortic injury. J Vasc Surg. 2016;63:1225–31. 26926941 10.1016/j.jvs.2015.11.046

[5] Kwolek CJ Blazick E. Current management of traumatic thoracic aortic injury. Semin Vasc Surg. 2010;23:215–20. 21194638 10.1053/j.semvascsurg.2010.11.003

[6] Tang GL Tehrani HY Usman A. Reduced mortality, paraplegia, and stroke with stent graft repair of blunt aortic transections: a modern meta-analysis. J Vasc Surg. 2008;47:671–5. 17980541 10.1016/j.jvs.2007.08.031

[7] Lee WA Matsumura JS Mitchell RS. Endovascular repair of traumatic thoracic aortic injury: clinical practice guidelines of the Society for Vascular Surgery. J Vasc Surg. 2011;53:187–92. 20974523 10.1016/j.jvs.2010.08.027

[8] Hagan PG Nienaber CA Isselbacher EM. The International Registry of Acute Aortic Dissection (IRAD): new insights into an old disease. JAMA. 2000;283:897–903. 10685714 10.1001/jama.283.7.897

[9] Mazzaccaro D Righini P Fancoli F. Blunt thoracic aortic injury. J Clin Med. 2023;12:2903. 37109240 10.3390/jcm12082903 PMC10142366

[10] Burks JJ Faries PL Gravereaux EC Hollier LH Marin ML. Endovascular repair of thoracic aortic aneurysms: stent-graft fixation across the aortic arch vessels. Ann Vasc Surg. 2002;16:24–8. 11904800 10.1007/s10016-001-0125-5

[11] Matsumura JS Rizvi AZ; Society for Vascular Surgery. Left subclavian artery revascularization: Society for Vascular Surgery Practice Guidelines. J Vasc Surg. 2010;52:65S–70S. 10.1016/j.jvs.2010.07.003 20875614

[12] Dinh K Limmer A Ngai C Cho T Young N Hsu J. Blunt thoracic aorta injuries, an Australian single centre’s perspective. ANZ J Surg. 2021;91:662–7. 33506996 10.1111/ans.16601

[13] Cindy M Sabrina H Kim D Geert M Inge F. Traumatic aortic rupture: 30 years of experience. Ann Vasc Surg. 2011;25:474–80. 21549915 10.1016/j.avsg.2010.12.019

[14] Arthurs ZM Starnes BW Sohn VY Singh N Martin MJ Andersen CA. Functional and survival outcomes in traumatic blunt thoracic aortic injuries: an analysis of the National Trauma Databank. J Vasc Surg. 2009;49:988–94. 19341888 10.1016/j.jvs.2008.11.052

[15] Fattori R Russo V Lovato L Di Bartolomeo R. Optimal management of traumatic aortic injury. Eur J Vasc Endovasc Surg. 2009;37:8–14. 19008125 10.1016/j.ejvs.2008.09.024

[16] Richens D Field M Neale M Oakley C. The mechanism of injury in blunt traumatic rupture of the aorta. Eur J Cardiothorac Surg. 2002;21:288–93. 11825737 10.1016/s1010-7940(01)01095-8

[17] Ho XN Wee IJ Syn N Harrison M Wilson L Choong AM. The endovascular repair of blunt traumatic thoracic aortic injury in Asia: a systematic review and meta-analysis. Vascular. 2019;27:213–23. 30739602 10.1177/1708538119828887

[18] Ungar TC Wolf SJ Haukoos JS Dyer DS Moore EE. Derivation of a clinical decision rule to exclude thoracic aortic imaging in patients with blunt chest trauma after motor vehicle collisions. J Trauma. 2006;61:1150–5. 17099521 10.1097/01.ta.0000239357.68782.30

[19] Fox N Schwartz D Salazar JH. Evaluation and management of blunt traumatic aortic injury: a practice management guideline from the Eastern Association for the Surgery of Trauma. J Trauma Nurs. 2015;22:99–110. 25768967 10.1097/JTN.0000000000000118

[20] Erbel R Aboyans V Boileau C. 2014 ESC guidelines on the diagnosis and treatment of aortic diseases: document covering acute and chronic aortic diseases of the thoracic and abdominal aorta of the adult. The Task Force for the Diagnosis and Treatment of Aortic Diseases of the European Society of Cardiology (ESC). Eur Heart J. 2014;35:2873–926. 25173340 10.1093/eurheartj/ehu281

[21] Cinnella G Dambrosio M Brienza N Tullo L Fiore T. Transesophageal echocardiography for diagnosis of traumatic aortic injury: an appraisal of the evidence. J Trauma. 2004;57:1246–55. 15625457 10.1097/01.ta.0000133576.35999.00

[22] Dahal R Acharya Y Tyroch AH Mukherjee D. Blunt thoracic aortic injury and contemporary management strategy. Angiology. 2022;73:497–507. 34990310 10.1177/00033197211052131

[23] Jang MO Kim JH Oh SK. Endovascular stent in traumatic thoracic aortic dissection. Korean Circ J. 2012;42:341–4. 22701500 10.4070/kcj.2012.42.5.341 PMC3369966

[24] Finkelmeier BA Mentzer RJ Kaiser DL Tegtmeyer CJ Nolan SP. Chronic traumatic thoracic aneurysm. Influence of operative treatment on natural history: an analysis of reported cases, 1950–1980. J Thorac Cardiovasc Surg. 1982;84:257–66. 7098511

[25] Passaro EJ Pace WG. Traumatic rupture of the aorta. Surgery. 1959;46:787–91. 14430738

[26] Khoynezhad A Donayre CE Azizzadeh A White R; RESCUE investigators. One-year results of thoracic endovascular aortic repair for blunt thoracic aortic injury (RESCUE trial). J Thorac Cardiovasc Surg. 2015;149:155–61.e4. 25439771 10.1016/j.jtcvs.2014.09.026

[27] Santanello SA Starr JE Larosa JL Barboza R Mcgee D Hari J. Traumatic rupture of the aorta: repair with a covered endovascular stent. J Trauma. 2002;52:1223. 12045659 10.1097/00005373-200206000-00035

[28] Kasirajan K Heffernan D Langsfeld M. Acute thoracic aortic trauma: a comparison of endoluminal stent grafts with open repair and nonoperative management. Ann Vasc Surg. 2003;17:589–95. 14569431 10.1007/s10016-003-0066-2

[29] Dubose JJ Leake SS Brenner M. Contemporary management and outcomes of blunt thoracic aortic injury: a multicenter retrospective study. J Trauma Acute Care Surg. 2015;78:360–9. 25757123 10.1097/TA.0000000000000521

[30] Mcbride CL Dubose JJ Miller CR. Intentional left subclavian artery coverage during thoracic endovascular aortic repair for traumatic aortic injury. J Vasc Surg. 2015;61:73–9. 25080884 10.1016/j.jvs.2014.05.099

[31] Ergun O Canyiğit M Hidiroğlu M. Endovascular treatment for acute traumatic thoracic aortic transection. Ulusal Travma Acil Cerrahi Derg. 2015;21:285–90. 10.5505/tjtes.2015.21556 26374416

[32] Mansour MA Kirk JS Cuff RF. Endovascular repair of traumatic thoracic aortic tears. Am J Surg. 2012;203:401–4;discussion404. 22265092 10.1016/j.amjsurg.2011.10.008

